# Supplementary material for: Ex Vivo Functional Assay for Evaluating Treatment Response in Tumor Tissue of Head and Neck Squamous Cell Carcinoma
Source: Cancers (Basel). 2023 Jan 12;15(2):478. doi: 10.3390/cancers15020478 (PMC9856585; doi:10.3390/cancers15020478)
Supplement: Supplementary file 1 [file cancers-15-00478-s001.zip › cancers-2101043-SI.pdf]

cancers

MDPI

Table S1. Composition of culture media used in the optimization of culture conditions of HNSCC tumor slices. DMEM: Dulbecco modified Eagle's medium; FCS: fetal calf serum; P/S: penicillin and streptomycin; B27: B-27 supplement; EGF: epidermal growth factor; bFGF: basic fibroblast growth factor; aDMEM: advanced DMEM/F-12.

Medium	Composition
**OC1**	
No primocin	DMEM + 10% FCS + P/S
	DMEM + 10% FCS + P/S + B27 50x; EGF 20ng/ml; insulin 1:1000
Primocin	DMEM + 10% FCS + P/S + primocin
	DMEM + 10% FCS + P/S + B27 50x; EGF 20ng/ml; insulin 1:1000 + primocin
**OC 2 - 8**	
Medium 1	DMEM + 0.5% BSA + P/S + primocin
Medium 2	DMEM + 0.5% BSA + P/S + B27 50x; EGF 40ng/ml; insulin 1:1000 + primocin
Medium 3	DMEM + 10% FCS + P/S + primocin
Medium 4	DMEM + 10% FCS + P/S + B27 50x; EGF 40ng/ml; insulin 1:1000 + primocin
**OC 7 - 14**	
Medium 5	aDMEM + P/S + primocin
Medium 6	aDMEM + P/S + B27 50x; EGF 20ng/ml; bFGF 20ng/ml; insulin 1:1000 + primocin
Medium 7	aDMEM + P/S + EGF 20ng/ml; bFGF 20ng/ml + primocin

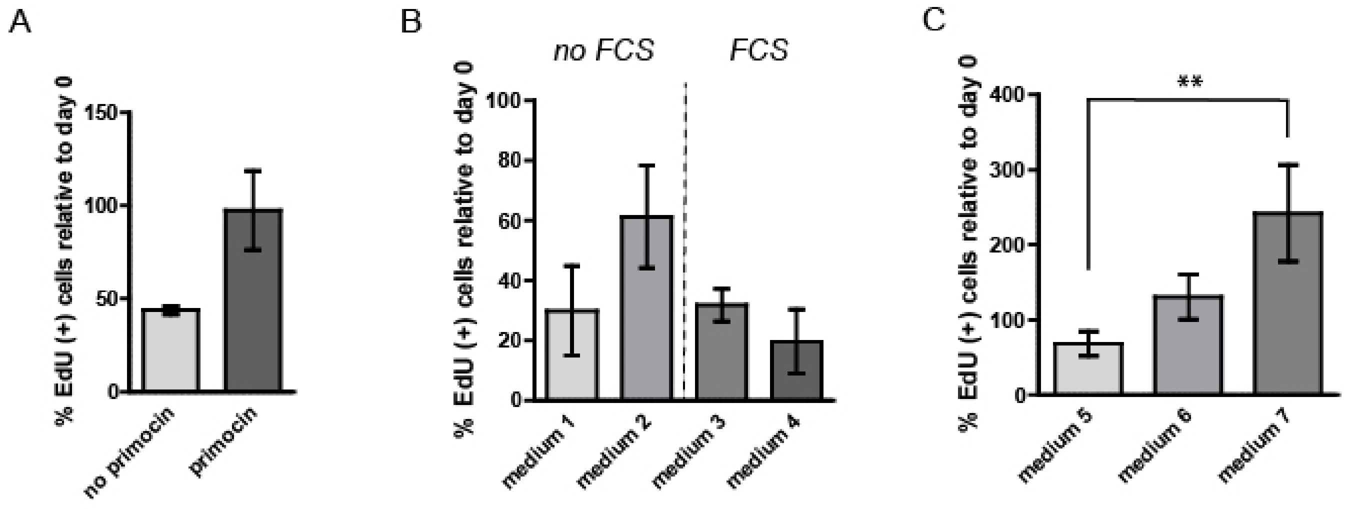

Figure S1. The effect of various culture media on the preservation of proliferation within HNSCC tumor slices during five days of culture. (**A**) Quantification of EdU positive cells in slices cultured in a medium with or without primocin. Bars show the mean of all slices (≥3) cultured in a given medium and error bars indicate SEM. (**B**) Quantification of EdU positive cells in slices cultured in a medium with or without FCS. Bars show the mean of all slices (≥3) cultured in a given medium and error bars indicate SEM. (**C**) Quantification of EdU positive cells in slices cultured in various aDMEM-based media. Bars show the mean of all slices (≥3) cultured in a given medium and error bars indicate SEM. The exact medium composition can be found in Table S1. t-test has been used to assess significance. ** $p < 0.001$.

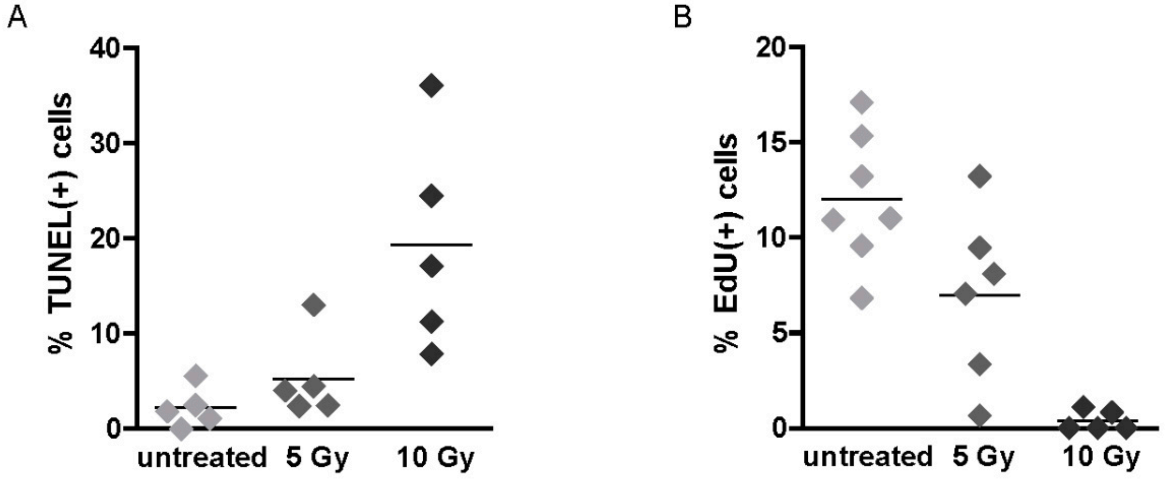

Figure S2. Irradiation with a single dose of 10 Gy induced marked apoptotic response and abolished proliferation in HNSCC tumor cells. (**A**) Quantification of TUNEL positive cells in untreated, and 5 Gy- or 10 Gy-treated samples at day 5 of the ex vivo culture. Each point represents the mean of ≥ 5 FoV per sample, and a mean across all samples per condition is indicated with a horizontal line. (**B**) Quantification of EdU positive cells in untreated, and 5 Gy- or 10 Gy-treated samples at day 5 of the ex vivo culture. Each point represents the mean of ≥ 5 FoV per sample, and a mean across all samples per condition is indicated with a horizontal line.
